# Novel diversity of polar Cyanobacteria revealed by genome-resolved metagenomics

**DOI:** 10.1099/mgen.0.001056

**Published:** 2023-07-07

**Authors:** Igor S. Pessi, Rafael V. Popin, Benoit Durieu, Yannick Lara, Bjorn Tytgat, Valentina Savaglia, Beatriz Roncero-Ramos, Jenni Hultman, Elie Verleyen, Wim Vyverman, Annick Wilmotte

**Affiliations:** ^1^​ Department of Microbiology, University of Helsinki, Helsinki, Finland; ^2^​ Helsinki Institute of Sustainability Science (HELSUS), Helsinki, Finland; ^3^​ InBioS – Centre for Protein Engineering, University of Liège, Liège, Belgium; ^4^​ Early Life Traces & Evolution-Astrobiology, UR-Astrobiology, University of Liège, Liège, Belgium; ^5^​ Laboratory of Protistology & Aquatic Ecology, Ghent University, Ghent, Belgium; ^6^​ Department of Plant Biology and Ecology, University of Sevilla, Sevilla, Spain; ^7^​ Natural Resources Institute Finland (LUKE), Helsinki, Finland

**Keywords:** Cyanobacteria, metagenomics, microbial mats, polar regions

## Abstract

Benthic microbial mats dominated by Cyanobacteria are important features of polar lakes. Although culture-independent studies have provided important insights into the diversity of polar Cyanobacteria, only a handful of genomes have been sequenced to date. Here, we applied a genome-resolved metagenomics approach to data obtained from Arctic, sub-Antarctic and Antarctic microbial mats. We recovered 37 metagenome-assembled genomes (MAGs) of Cyanobacteria representing 17 distinct species, most of which are only distantly related to genomes that have been sequenced so far. These include (i) lineages that are common in polar microbial mats such as the filamentous taxa *Pseudanabaena *, *Leptolyngbya *, *Microcoleus */* Tychonema* and *Phormidium*; (ii) the less common taxa *Crinalium* and *Chamaesiphon *; (iii) an enigmatic *Chroococcales* lineage only distantly related to *Microcystis *; and (iv) an early branching lineage in the order *Gloeobacterales* that is distributed across the cold biosphere, for which we propose the name *Candidatus* Sivonenia alaskensis. Our results show that genome-resolved metagenomics is a powerful tool for expanding our understanding of the diversity of Cyanobacteria, especially in understudied remote and extreme environments.

## Data Summary

The sequencing data generated in this study have been submitted to the European Nucleotide Archive (ENA) under the BioProject PRJEB59431. Individual accession numbers for raw reads and genomic bins are listed in Tables S1 and S3, available in the online version of this article, respectively. Genomic bins can also be downloaded from doi.org/10.6084 /m9 .figshare.22003967[1]. The bioinformatics workflow used throughout this study is available in github.com/igorspp/polar-cyanobacteria-MAGs.

**Impact Statement**

Cyanobacteria are photosynthetic microorganisms that play important roles in polar lakes. Many Cyanobacteria are difficult to grow in the laboratory, particularly in isolation from other organisms, which makes it challenging to sequence their genomes. As such, considerably fewer genomes of Cyanobacteria have been sequenced so far compared to other bacteria. In this study, we used a metagenomics approach to recover novel genomes of Cyanobacteria from Arctic and Antarctic microbial mats without the need to isolate the organisms. The community DNA was extracted and sequenced, and the genomes of individual populations were separated using bioinformatics tools. We recovered the genomes of 17 different species of Cyanobacteria, many of which have not been sequenced before. We describe in more detail an interesting lineage of ancestral Cyanobacteria in the order *Gloeobacterales *, for which we propose the name *Candidatus* Sivonenia alaskensis. Our study shows that genome-resolved metagenomics is a valuable approach for obtaining novel genomes of Cyanobacteria, which are needed to improve our understanding of life in the polar regions and the planet at large.

## Introduction

Microbial mats are highly successful and productive systems found in a wide range of environments since the dawn of life on Earth [2, 3]. Microbial mats commonly comprise a vast diversity of microorganisms such as auto- and heterotrophic bacteria, fungi, microalgae, and heterotrophic protists embedded in an exopolysaccharide matrix [4]. Benthic microbial mats represent an important survival strategy against the harsh environmental conditions in polar and alpine lakes, and often have Cyanobacteria as their primary source of organic carbon and nitrogen [5, 6]. In addition to aquatic microbial mats, Cyanobacteria are also important members of terrestrial and epi- and supraglacial communities in polar environments [7, 8].

Despite their importance, knowledge on the diversity and ecology of Cyanobacteria in polar environments is fragmentary [9]. Studies on the diversity of polar Cyanobacteria have mostly focused on microscopic identification and strain isolation [10–16], analysis of environmental 16S rRNA gene sequences [17–22] or a combination of these methods [23–25]. On the one hand, the microscopic identification of Cyanobacteria is hindered by the high plasticity of taxonomic markers such as cell dimensions and division patterns and the relative paucity of morphological characters [26]. In addition, morphology-based assessments underestimate the diversity of Cyanobacteria in the environment compared to molecular approaches based on environmental DNA [20]. Molecular approaches, in turn, are hampered by the scarcity of cyanobacterial genomes stored in public databases, which are largely underrepresented compared to other microbial phyla and heavily biased towards the marine *Prochlorococcus */* Synechococcus* clade [27, 28].

The genomic catalogue of polar Cyanobacteria is currently limited to a handful of strains, including *Pseudanabaena* sp. BC1403 and *Phormidesmis priestleyi* BC1401 from Greenland [29], *Leptolyngbya* sp. Cla-17 from the Canadian Arctic [30], and the Antarctic strains *Phormidesmis priestleyi* ULC007 [31], *Leptolyngbya* sp. BC1307 [32], *Synechococcus* sp. SynAce01 [33] and *Nostoc* sp. SO-36 [34]. Twelve other low-quality genomes obtained by a metagenome-like assembly approach of non-axenic strains are also available [35]. Genome-resolved metagenomics has been established in recent years as a powerful approach to obtain microbial genomes, as it circumvents the difficulties associated with culturing microorganisms by reconstructing microbial genomes directly from environmental DNA [36–38]. Many genomes of uncultured polar Cyanobacteria have been obtained recently using this approach, including several novel lineages of early branching Cyanobacteria in the order *Gloeobacterales* [39–41].

In this study, we aimed to expand the genomic catalogue of polar Cyanobacteria. To achieve this, we applied a genome-resolved metagenomics approach to data obtained from microbial mats from Arctic, sub-Antarctic and Antarctic lakes spanning a wide geographical and limnological range. Our results include the recovery of novel genomes of polar Cyanobacteria and the description of an early branching lineage that is distributed across polar and alpine environments.

## Methods

### Description of samples and sampling procedure

**Fig. 1.**
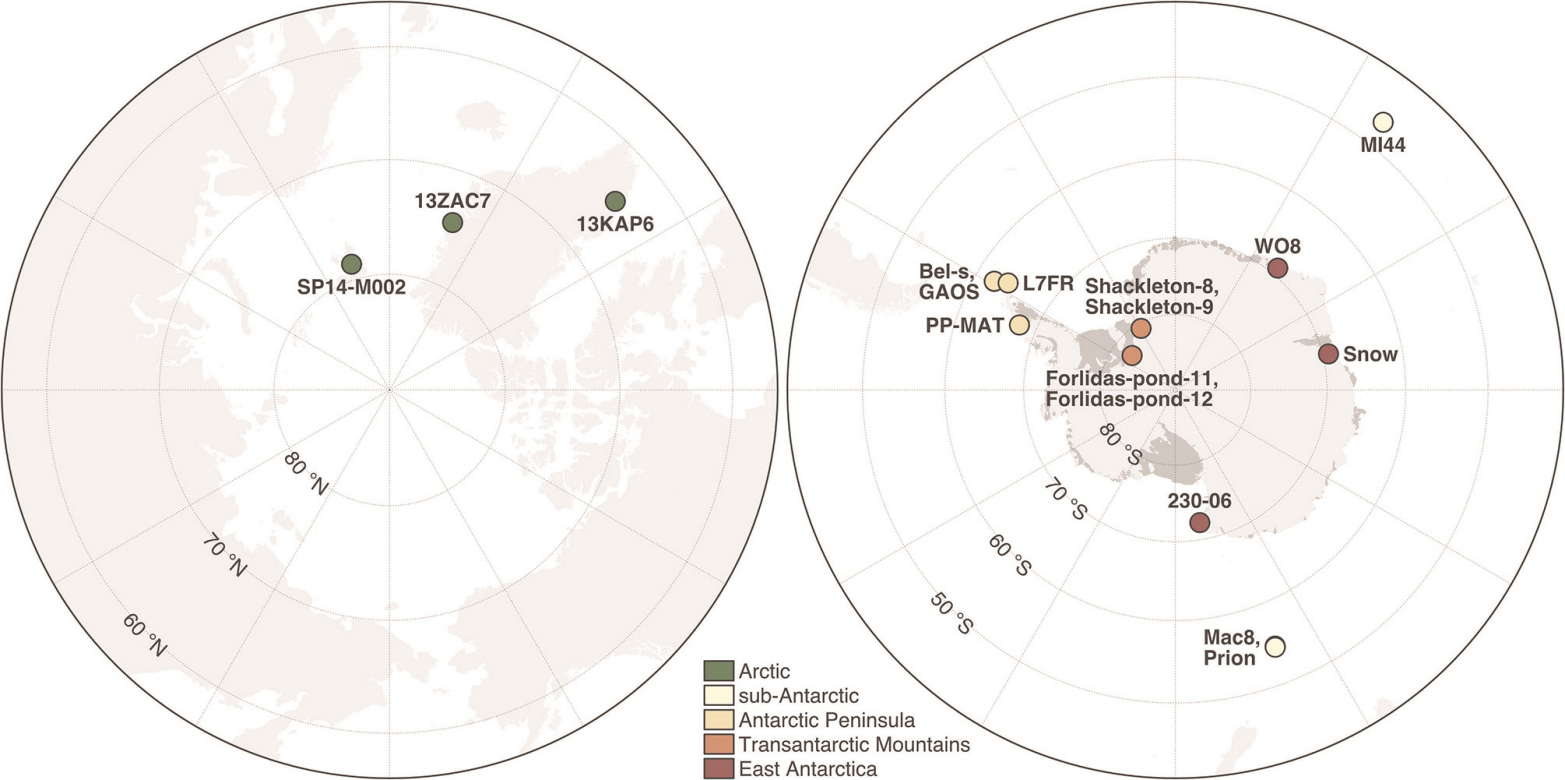
Location of the Arctic (left) and Antarctic (right) lakes where microbial mats were sampled for this study. Maps were created with public open access data from the Norwegian Polar Institute (Tromsø, Norway). More information about the samples can be found in Table S1.

We analysed 17 microbial mat samples obtained from 15 Arctic, sub-Antarctic and Antarctic lakes (Fig. 1). The studied lakes were selected from a larger set of 216 polar lakes (B. Tytgat *et al*., submitted) and encompass the main polar biogeographical regions, namely the Arctic (Greenland and Svalbard), sub-Antarctic (Macquarie Island in the South Pacific Ocean and Marion Island in the South Indian Ocean), Antarctic Peninsula, Transantarctic Mountains and East Antarctica. The Antarctic lakes are distributed across five Antarctic Conservation Biological Regions (ACBRs) [42]. We aimed to include in our sampling design at least three samples from each main biogeographical region. Furthermore, with the exception of lakes in the Transantarctic Mountains, the samples were selected based on comparable values of pH (7.4±0.8) and conductivity (0.2±0.1 mS cm^–1^) (Table S1), since these parameters are known to be major drivers of polar microbial community composition [43]. The pH and conductivity of the Transantarctic Mountains lakes are 8.1±0.6 and 36.9±60.9 mS cm^–1^, respectively. A macroscopic description of each sample can be found in Table S1.

The samples were taken following a standardized protocol [44, 45] in the course of several coordinated national and international limnological research programmes. In general, visible mats or biofilms were sampled in the littoral ice-free zone of the lakes (20–50 cm from the lake shore) using a sterilized spatula. Benthic microbial mats at depths >1 m in the Antarctic Peninsula were sampled using a Glew surface sediment corer, and the upper 1 cm of the core was aseptically collected using a sterilized spatula. In the Transantarctic Mountains, benthic samples were collected in the littoral (moat) zone below 10–15 cm of lake ice, as well as from the deepest part of the lakes after drilling through the ice using a Jiffy Drill. All samples were stored immediately in the dark and kept frozen at –20 °C until processing.

### DNA extraction and metagenome sequencing

We used the DNeasy PowerBiofilm DNA Isolation kit (Qiagen) to extract DNA from *ca*. 0.5 g of each microbial mat sample and checked the concentration and quality of the DNA extracts using the Qubit dsDNA BR Assay kit (Thermo Fisher Scientific). We used the Nextera XT kit (Illumina) to prepare the metagenomic libraries, which were then sent to Eurofins Genomics for sequencing using the Illumina HiSeq 2500 platform (2×100 bp). We checked the quality of the raw sequencing data with *fastQC* v0.11.9 (bioinformatics.babraham.ac.uk/projects/fastqc) and *multiQC* v1.8 [46], and used *Cutadapt* v1.16 [47] to trim adapters and low-quality base calls (Phred score <20), and to discard short reads (<50 bp). Finally, we used *METAXA* v2.2 [48] to extract reads matching the 16S rRNA gene, which were then classified with *mothur* v1.44.3 [49] using the silva database release 138.1 [50] and the Naïve Bayesian Classifier with a confidence cut-off of 80 % [51].

### Metagenome assembling and binning

We assembled and binned each metagenome individually and as two co-assemblies with *MEGAHIT* v1.1.1.2 [52]. One co-assembly was done by grouping the Arctic (*n*=3) and sub-Antarctic (*n*=3) samples. The second co-assembly comprised the remaining samples from the Antarctic Peninsula, Transantarctic Mountains and East Antarctica (*n*=11). For each individual and co-assembly, we used *anvi’o* v7.0 [53] to bin contigs ≥2500 bp into metagenome-assembled genomes (MAGs) as previously described [37, 38]. In brief, we used *Prodigal* v2.6.3 [54] to find gene calls, *HMMER* v.3.3 [55] to identify a set of 71 bacterial and 76 archaeal single-copy genes [56], and *DIAMOND* v0.9.14 [57] to assign taxonomy to the single-copy genes according to the Genome Taxonomy Database (GTDB) release 04-RS89 [58]. We used *bowtie* v2.4.2 [59] to map the quality-filtered reads from all samples to the contigs and *SAMtools* v1.1 [60] to sort and index the mapping output. We then used the *anvi-interactive* interface of *anvi’o* to manually sort the contigs into genomic bins based on differential coverage and tetranucleotide frequency. Bins that were ≥50 % complete according to the presence of single-copy genes [56] were manually curated using the *anvi-refine* interface of *anvi’o*. We refined the bins by removing outlying contigs according to coverage, tetranucleotide frequency and taxonomic signal of single-copy genes. We assigned taxonomy to the refined bins based on 122 archaeal and 120 bacterial single-copy genes with *GTDB-Tk* v1.3.0 [61] and the GTDB release 05-RS95 [58]. Bins assigned to the phylum *Cyanobacteria* that were ≥50 % complete and ≤10 % redundant – hereafter referred as MAGs – were kept for downstream analyses. We used *fastANI* v1.32 [62] to compute the genome-wide average nucleotide identity (ANI) between the Cyanobacteria MAGs. We considered that MAGs sharing ≥95 % ANI belong to the same species according to Konstantinidis *et al*. [63].

### Phylogenetic analysis

We used a concatenated alignment of 38 ribosomal proteins to place the MAGs in a phylogenetic tree alongside all genomes assigned to the Cyanobacteria/Melainabacteria group in GenBank (NCBI:txid1798711, accessed on 17 November 2022). We used *ncbi-genome-download* v0.3.1 (github.com/kblin/ncbi-genome-download) to recover the genomes from GenBank. In *anvi’o* v7.0 [53], we retrieved the translated amino acid sequence of each ribosomal protein with *HMMER* v3.3 [55] and aligned them with *muscle* v3.8.1551 [64]. We concatenated the alignments of the 38 ribosomal proteins and built a maximum-likelihood tree with *IQ-TREE* v2.1.4 [65] using the automatic model selection and 1000 ultrafast bootstrap approximation replicates. We also used *fastANI* v1.32 [62] to calculate the genome-wide ANI between MAGs and GenBank genomes. For better visualization, we computed a more compact maximum-likelihood tree including only the MAGs, their closest neighbours in GenBank, strains from the Pasteur Culture Collection of Cyanobacteria (PCC) and other selected genomes. We classified the MAGs based on their phylogenetic placement following the taxonomic system of Komárek *et al*. [66].

### Gene annotation

In *anvi’o* v7.0 [53], we annotated the gene calls identified by *Prodigal* v2.6.3 [54] against the KOfam [67] and the Pfam [68] databases with *HMMER* v3.3 [55] and the COG [69] database with *DIAMOND* v0.9.14 [57]. We also used *tblastn* (web interface, available at blast.ncbi.nlm.nih.gov/Blast.cgi?PROGRAM = tblastn and PAGE_TYPE = BlastSearch) to search for additional genes involved in mechanisms of resistance to stress. Only hits with an e-value <10^–5^ and bitscore >50 were considered, according to Pearson [70].

### Distribution analyses

We used metagenomic read recruitment to compute the relative abundance of each MAG across the 17 microbial mat samples. Prior to this, we used *dRep* v3.2.2 [71] to dereplicate the MAGs based on a ≥99 % ANI threshold. We then used *CoverM* v0.6.1 (github.com/wwood/CoverM) to map the quality-filtered reads to the MAGs with *minimap* v2.17 [72] and compute relative abundances based on the proportion of reads recruited by the MAGs. For this, we considered only matches with ≥95 % identity and ≥75 % coverage. We also used *sourmash branchwater* [73, 74] and *IMNGS* [75] to search the two *Gloeobacterales* MAGs against metagenomic and amplicon sequencing datasets in the Sequence Read Archive (SRA), respectively. For the first, we used the *mastiff* implementation of *sourmash branchwater* (github.com/sourmash-bio/2022-search-sra-with-mastiff). The datasets where significant matches were found (containment ≥20 %) were downloaded from SRA with *fasterq-dump* v3.0.1 (github.com/ncbi/sra-tools) and mapped back to the two *Gloeobacterales* MAGs with *CoverM* v0.6.1 as described above. For the analysis of amplicon sequencing datasets, we used the web interface of *IMNGS* (imngs.org) and only considered datasets where significant matches (≥99 % similarity) accounted for ≥0.1 % of the sequences.

## Results and discussion

### Taxonomic profiling of the microbial mat communities

We obtained around 500 million paired-end metagenomic reads (99.3 Gb) from 17 Arctic, sub-Antarctic and Antarctic microbial mat samples (Fig. 1, Table S1). Taxonomic profiling based on reads matching the 16S rRNA gene revealed *Cyanobacteria* as the second most abundant microbial phylum after *Proteobacteria* (mean relative abundance of 20.8 and 24.0 %, respectively) (Table S2). The dominance of these two phyla is commonly observed in polar microbial mats [76–78]. However, we observed differences in taxonomic composition across the samples (Table S2). For example, Cyanobacteria made up 63.5 % of the microbial community in the sample ‘Forlidas-pond-12’, a microbial mat taken from the hypersaline brine layer at the bottom of Forlidas Pond (Transantarctic Mountains) which has been known to harbour relatively simple communities [20, 21, 25]. On the other hand, Cyanobacteria were virtually absent in samples ‘Prion’ (sub-Antarctic), ‘GAOS’ (Antarctic Peninsula), ‘Shackleton-9’ (Transantarctic Mountains) and ‘WO8’ (East Antarctica). Rather than being related to geographical and climatic aspects of the sampling sites, the observed differences in taxonomic composition probably reflect the heterogenous characteristics of the microbial mats analysed in this study (see Table S1 for a macroscopic description of the samples). Although our sampling design includes a diverse set of Arctic, sub-Antarctic and Antarctic lakes, it is evident that we have covered only a minute fraction of the entire limnological and biological diversity of polar and sub-polar regions.

### Recovery of cyanobacterial genomes from metagenomic data

**Fig. 2.**
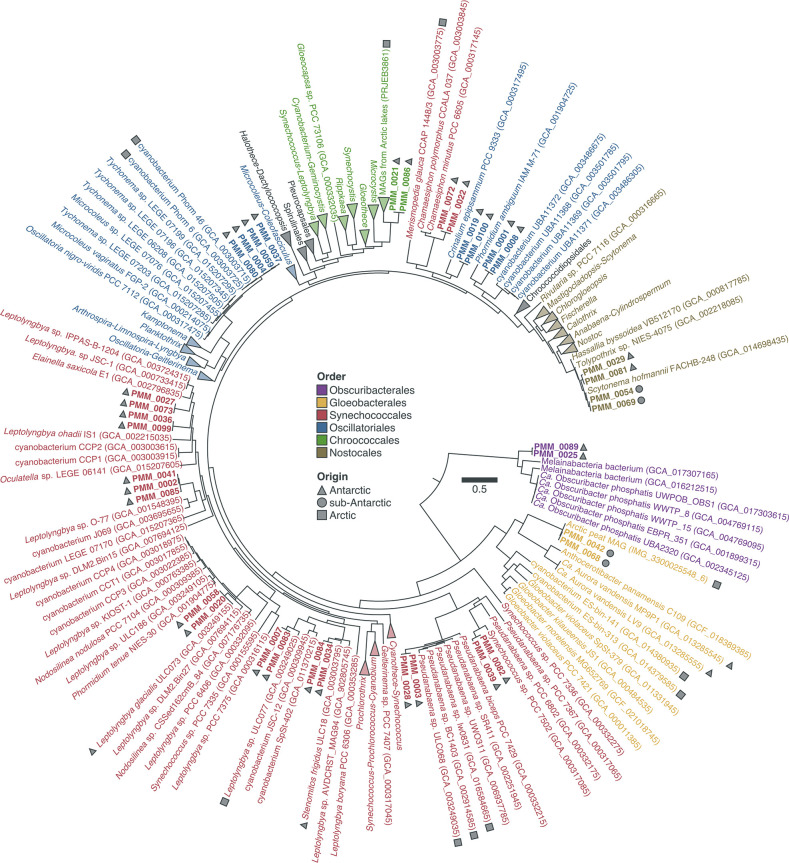
Phylogenetic analysis of 37 metagenome-assembled genomes (MAGs) assigned to the phylum *Cyanobacteria *, including both Cyanobacteria *stricto sensu* (clade Oxyphotobacteria) and the Melainabacteria. Maximum-likelihood tree (LG+R8 model) based on a concatenated alignment of 38 ribosomal proteins from the MAGs (in bold), their closest neighbours in GenBank, PCC strains and other selected genomes. The geographical origins of polar MAGs and strains are indicated. Order-level classification is shown according to the taxonomic system of Komárek *et al*. [66]. The scale bar indicates the number of amino acid changes per site.

We assembled the metagenomes with *MEGAHIT* [52] and obtained 176 097 and 72 514 contigs ≥1000 bp for the Antarctic and Arctic/sub-Antarctic co-assemblies, respectively. The total assembled length was 447.6 and 182.2 Mb, respectively. The output of the individual assemblies ranged from 218 contigs/0.3 Mb (sample ‘13ZAC7’) to 96 722 contigs/262.4 Mb (sample ‘PP-MAT’) (Table S1). The assembly of sample ‘Forlidas-pond-11’ did not yield any contigs due to the very low sequencing depth achieved for this sample. After assembling the metagenomes, we used *anvi’o* [53] to manually bin and curate MAGs. Taxonomic classification based on the GTDB release 05-RS95 [58] assigned 37 MAGs to the phylum *Cyanobacteria* (Fig. 2, Table 1). Most MAGs originated from the individual assemblies of Antarctic samples (*n*=20) and the Antarctic co-assembly (*n*=15) (Table S3). We did not recover any MAG from the individual assemblies of Arctic samples despite the high abundance of Cyanobacteria in these samples (5.9–28.6 % of the reads matching the 16S rRNA gene) (Table S2) and the high sequencing depth (3.6–7.5 Gb) (Table S1). The disparity in the number of MAGs recovered from each sample is probably related to differences in sequencing depth which, together with the level of complexity of the underlying communities, affect the number of genomes that are sufficiently represented in a metagenomic dataset [79].

**Table 1.** Information on 37 metagenome-assembled genomes (MAGs) of Cyanobacteria *stricto sensu* (clade Oxyphotobacteria) and Melainabacteria recovered from polar microbial mats.

Group	MAG	Species cluster*	Size (Mb)	Completion (%)†	Redundancy (%)†	GC (%)
* Obscuribacterales *	PMM_0025	sp.1	6.1	90.1	8.5	47.7
	PMM_0089	sp.1	4.7	77.5	5.6	47.9
* Gloeobacterales *	PMM_0042	sp.2	2.9	97.2	0	49.3
	PMM_0068	sp.2	2.8	95.8	0	48.8
* Synechococcales *	PMM_0039	sp.3	1.5	57.7	0	41.5
	PMM_0082	sp.3	2.7	85.9	0	40.3
	PMM_0003	sp.4	3.1	78.9	5.6	42.9
	PMM_0028	sp.4	3.7	84.5	8.5	42.6
	PMM_0034	sp.5	3.3	66.2	0	51.3
	PMM_0084	sp.5	4.8	81.7	4.2	51.8
	PMM_0007	sp.6	5.2	94.4	1.4	49
	PMM_0083	sp.6	4.6	94.4	1.4	48.4
	PMM_0020	sp.7	3.2	90.1	2.8	57.2
	PMM_0058	sp.7	2.8	84.5	1.4	56.9
	PMM_0002	sp.8	2.9	90.1	1.4	52.5
	PMM_0041	sp.8	2.5	50.7	1.4	52.4
	PMM_0085	sp.8	3.7	81.7	4.2	52.5
	PMM_0036	sp.9	4.6	66.2	4.2	49.4
	PMM_0099	sp.9	4.7	67.6	5.6	49.3
	PMM_0027	sp.10	2.2	73.2	1.4	55
	PMM_0073	sp.10	3.6	85.9	5.6	55.3
	PMM_0022	sp.11	2.5	80.3	2.8	44.9
	PMM_0072	sp.11	2.9	83.1	1.4	44.4
* Oscillatoriales *	PMM_0004	sp.12	4.4	60.6	7	45.6
	PMM_0037	sp.12	5.2	67.6	8.5	45.3
	PMM_0059	sp.12	4.9	90.1	5.6	45.6
	PMM_0080	sp.12	6.4	94.4	2.8	45.1
	PMM_0019	sp.13	5.7	95.8	2.8	45.2
	PMM_0100	sp.13	3.8	80.3	2.8	45.4
	PMM_0001	sp.14	5.8	94.4	2.8	45.4
	PMM_0008	sp.14	5.8	94.4	2.8	45.4
* Chroococcales *	PMM_0021	sp.15	4	77.5	2.8	39
	PMM_0086	sp.15	3.8	80.3	2.8	38.9
* Nostocales *	PMM_0029	sp.16	2.7	73.2	7	42
	PMM_0081	sp.16	4.4	91.5	2.8	42.1
	PMM_0054	sp.17	5.1	90.1	2.8	42.2
	PMM_0069	sp.17	3	76.1	0	41.6

*MAGs in the same species cluster share ≥95% average nucleotide identity (ANI).

†Completion and redundancy levels were estimated based on the presence of 71 single-copy genes [56] with *anvi’o* [53]. More information about the MAGs can be found in Table S3.

**Fig. 3.**
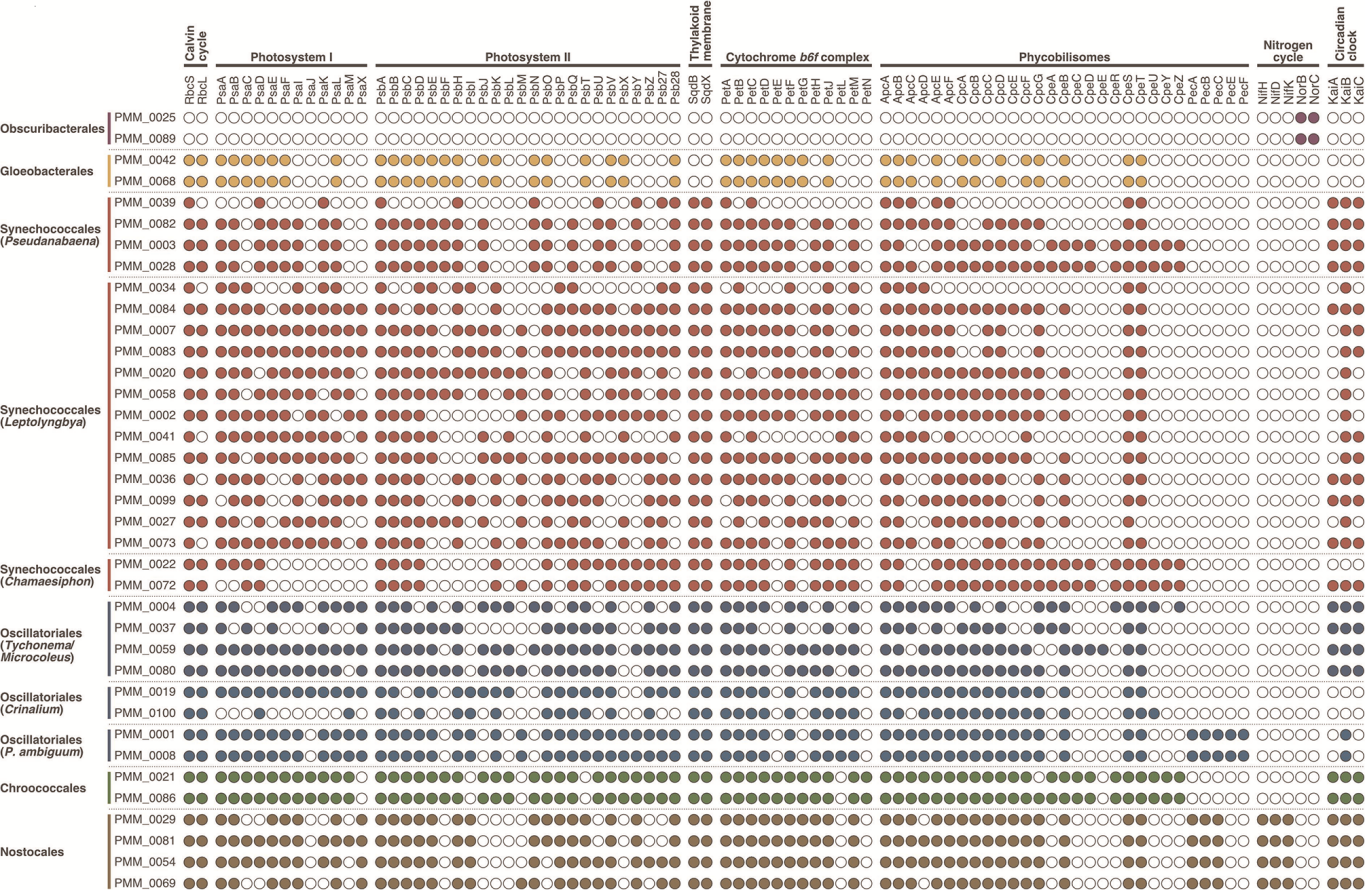
Presence of genes involved in carbon fixation, photosynthesis, nitrogen cycle and circadian clock in 37 metagenome-assembled genomes (MAGs) of Cyanobacteria *stricto sensu* (clade Oxyphotobacteria) and Melainabacteria.

MAG dereplication based on a ≥95 % ANI threshold [63] grouped the 37 Cyanobacteria MAGs into 17 species-level clusters (Table 1). Phylogenetic analysis based on a concatenated alignment of 38 ribosomal proteins assigned 35 of the 37 Cyanobacteria MAGs to the clade Oxyphotobacteria (Cyanobacteria *stricto sensu*) (Fig. 2, Table 1). These belong to five orders according to the taxonomic system of Komárek *et al*. [66]: *Gloeobacterales* (*n*=2), *Synechococcales* (*n*=19), *Oscillatoriales* (*n*=8), *Chroococcales* (*n*=2) and *Nostocales* (*n*=4). The other two MAGs belong to the order *Obscuribacterales* of the Melainabacteria, a sister lineage to the Oxyphotobacteria that lacks the photosynthetic machinery [80]. Indeed, annotation of protein-coding genes revealed that the two *Obscuribacterales* MAGs do not appear to encode proteins of the Calvin cycle (Rbc), photosystems I and II (Psa and Psb), cytochrome *b6f* complex (Pet), or phycobilisomes (Apc, Cpc, Cpe, and Pec) (Fig. 3). Interestingly, the presence of genes for the small and large subunits of nitric oxide reductase (NorC and NorB, respectively) indicates a potential role of this lineage in the production of the greenhouse gas nitrous oxide [37].

### Genome-resolved metagenomics is a reliable tool for the investigation of cyanobacterial diversity

In general, we observed a good correspondence between individual and co-assembly MAGs, that is, closely related genomic bins with ≥99 % ANI were recovered from the two assembly types (Table S3). The robustness of our metagenomic approach is further illustrated by the high similarity that some of the MAGs share with genomes available in GenBank. In particular, two MAGs obtained from different assemblies (‘PMM_0058’ and ‘PMM_0020’) are almost identical (99.7–99.8% ANI) to the genome of the strain *Leptolyngbya glacialis* ULC073 (Fig. 2, Table S3). This is not surprising given that the three genomes originate from the same hypersaline brine layer in the benthos of Forlidas Pond, Transantarctic Mountains [25]. *Leptolyngbya glacialis* ULC073 and *Leptolyngbya antarctica* ULC047 (Ace Lake, Princess Elizabeth Land) [13], which share identical 16S rRNA gene sequences, are representative strains of an ubiquitous morpho-/genotype in Antarctic lakes belonging to the *Leptolyngbya–Nodosilinea* clade [13, 21, 24, 25]. Despite the importance of this lineage, the genome of *Leptolyngbia glacialis* ULC073 currently available in GenBank (accession GCA_003249155.1), which was obtained from a non-axenic unialgal culture using a metagenome-like approach [35], is very fragmented (650 contigs, *N*_50_=10.7 kb) and somewhat redundant (7.0 % according to our analysis of 71 single-copy genes). Based on these parameters, the MAGs ‘PMM_0058’ and ‘PMM_0020’, which are 84.5–90.1% complete, 1.4–2.8% redundant, comprise 290–339 contigs and have an *N*_50_ of 12.1–13.0 kb (Tables 1 and S3), can be considered better representatives of this important lineage of Antarctic Cyanobacteria.

Other MAGs that are closely related to strains are the *Nostocales* MAGs ‘PMM_0054’ and ‘PMM_0069’. Genome-wide analysis revealed that they share 93.8–94.5% ANI with their closest genome on GenBank, *Scytonema hofmannii* FACHB-248 (Fig. 2, Table S3). However, their 16S rRNA gene is 99.4 % similar to the sequence of *Dactylothamnos antarcticus* CENA433 isolated from a freshwater biofilm in the Antarctic Peninsula [81], for which genomic information is currently lacking. Finally, the *Gloeobacterales* MAGs ‘PMM_0042’ and ‘PMM_0068’ share 97.2 % ANI with the MAG ‘IMG_3300025548_6’ recovered from peat soil in Alaska [39] (Fig. 2, Table S3).

### Metagenomics reveals novel genomic diversity of polar Cyanobacteria

Phylogenetic placement and genome-wide comparison with sequences from GenBank revealed that most MAGs differ from genomes that have been sequenced so far (Fig. 2, Table S3). In particular, 19 of the 37 MAGs have <80 % ANI with genomes currently available in GenBank and 12 are only distantly related to existing genomes (80.1–93.2% ANI) (Table S3). Interestingly, phylogenetic placement clustered 16 and eight MAGs alongside polar and alpine strains, respectively (Fig. 2). This is in agreement with previous studies showing that many lineages of Cyanobacteria are distributed across the cold biosphere [21, 82, 83]. Most MAGs are affiliated with filamentous taxa in the orders *Synechococcales* (*n*=17), *Oscillatoriales* (*n*=8) and *Nostocales* (*n*=4) (Fig. 2, Table 1), highlighting the importance of filamentous Cyanobacteria as the builders of polar microbial mat ecosystems [5, 6, 84, 85]. Moreover, Cyanobacteria belonging to the order *Nostocales* often dominate the microbial communities in oligotrophic polar environments due to their ability to fix atmospheric nitrogen [5–8]. As observed previously (e.g. Olson *et al*. [86]), genes encoding the different subunits of the nitrogenase enzyme (NifHDK) involved in nitrogen fixation were exclusive to the four *Nostocales* MAGs (Fig. 3).

Most *Synechococcales* MAGs (*n*=13) are phylogenetically related to strains that have been traditionally classified as *Leptolyngbya *, which is a morphological group comprising Cyanobacteria with a thin, simple filamentous morphotype that includes many different genera according to molecular data [28, 66]. Our *Leptolyngbya* MAGs can be broadly categorized into four major lineages (Fig. 2): (i) *Leptolyngbya stricto sensu* (‘PMM_0007’ and ‘PMM_0083’), (ii) *Leptolyngbya–Stenomitos* (‘PMM_0034’ and ‘PMM_0084’), (iii) *Leptolyngbya–Nodosilinea* (‘PMM_0020’ and ‘PMM_0058’), and (iv) *Leptolyngbya–Oculatella–Elainella* (‘PMM_0085’, ‘PMM_0002’, ‘PMM_0041’, ‘PMM_0099’, ‘PMM_0036’, ‘PMM_0073’ and ‘PMM_0027’). The other four MAGs of filamentous *Synechococcales* are affiliated with the early branching *Pseudanabaena* (Fig. 2). Two of these (‘PMM_0003’ and ‘PMM_0028’) are most closely related (80.9–81.0% ANI) to the strain *Pseudanabaena* sp. ULC068 isolated from a lake in the Canadian sub-Arctic (W. Vincent, unpublished) (Table S3), and also clustered alongside the strains BC1403 from Greenland [29] and lw0831 from Svalbard [87] (Fig. 2). The other two *Pseudanabaena* MAGs (‘PMM_0039’ and ‘PMM_0082’) are distantly related (<80 % ANI) to *Synechococcus* sp. PCC 7502, a unicellular strain isolated from an alpine *Sphagnum* bog that clusters with the early branching *Pseudanabaena* [27].

Other MAGs of filamentous Cyanobacteria are affiliated with the order *Oscillatoriales* (*n*=8) (Fig. 2). Four of these (‘PMM_0004’, ‘PMM_0037’, ‘PMM_0059’ and ‘PMM_0080’) are most closely related (90.1–93.2% ANI) to the strain Phorm 46 isolated from a lake in the Canadian Arctic [88] (Table S3), and also clustered alongside strains of *Tychonema* and *Microcoleus vaginatus* (Fig. 2). The other two MAGs of filamentous *Oscillatoriales* (‘PMM_0001’ and ‘PMM_0008’) are distantly related (<80 % ANI) to the strain *Phormidium ambiguum* IAM M-71, which has an uncertain phylogenetic placement. Phylogenetic analysis of the amplified 16S rRNA gene sequence (accession AB003167) originally placed *Phormidium ambiguum* IAM M-71 alongside other *Oscillatoriales* such as *Oscillatoria* and *Lyngbya* [89, 90]. However, a later 16S rRNA phylogeny [91] and a phylogenomic tree based on 834 single-copy genes [92] both placed the strain IAM M-71 in a similar phylogenetic position as the one inferred here, that is, basal to the *Nostocales* (Fig. 2). A blast analysis suggests that the AB003167 sequence is chimeric with *Phormidium muscicola* IAM M-221, but a phylogenetic artefact based on long branch attraction is also possible given the lack of related genomes. Interestingly, the *P. ambiguum* MAGs were the most widespread MAGs in our dataset, being detected in five samples in the Antarctic Peninsula, Transantarctic Mountains and East Antarctica (Table S4). Finally, the two remaining *Oscillatoriales* MAGs (‘PMM_0019’ and ‘PMM_0100’) clustered alongside *Crinalium epipsammum* PCC 9333 (Fig. 2). *Crinalium* is a filamentous genus of Cyanobacteria with unusual elliptical trichomes [93]. Sequences related to *Crinalium* have been recovered from different alpine habitats [94] and a new species, *Crinalium glaciale*, has been described from cryoconite pools in Antarctica on the basis of morphological identification [95].

In addition to filamentous taxa, we also recovered MAGs related to unicellular Cyanobacteria in the orders *Gloeobacterales* (*n*=2), *Synechococcales* (*n*=2) and *Chroococcales* (*n*=2) (Fig. 2). All except the two *Gloeobacterales* MAGs were distantly related (<80 % ANI) to genomes currently available in GenBank (Table S3). The two *Synechococcales* MAGs (‘PMM_0022’ and ‘PMM_0072’) clustered alongside *Chamaesiphon minutus* PCC 6605 and *Chamaesiphon polymorphus* CCALA 037 (Fig. 2). *Chamaesiphon* is a cosmopolitan genus that is often reported in polar and alpine terrestrial and aquatic environments, and includes two species potentially endemic to Antarctica (*Chamaesiphon arctowskii* and *Chamaesiphon austro-polonicus*) [16, 20, 21, 84, 85, 96]. Finally, the two *Chroococcales* MAGs (‘PMM_0021’ and ‘PMM_0086’) formed a distinct lineage related to *Microcystis* and several MAGs recovered from Arctic lakes (BioProject PRJEB38681) (Fig. 2).

### Description of *Candidatus* Sivonenia alaskensis

**Fig. 4.**
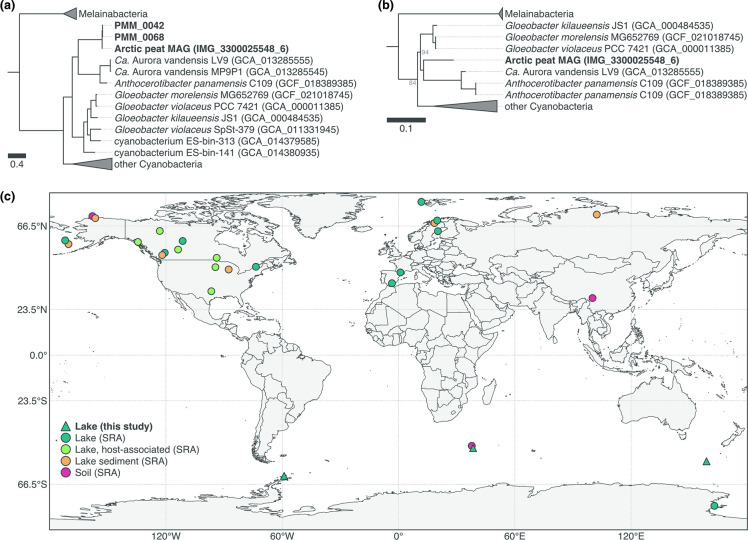
*Candidatus* Sivonenia alaskensis, a lineage of early branching Cyanobacteria in the order *Gloeobacterales *. (**a**) Maximum-likelihood tree (LG+R8 model) based on a concatenated alignment of 38 ribosomal proteins from the three *Ca*. Sivonenia alaskensis MAGs (in bold) and other *Gloeobacterales* and selected genomes from GenBank. All nodes have bootstrap support ≥95 %. The scale bar indicates the number of amino acid changes per site. (b) Maximum-likelihood tree (GTR+F+R8 model) of the 16S rRNA gene of *Ca*. Sivonenia alaskensis. Nodes have bootstrap support ≥95 % unless shown otherwise. The scale bar indicates the number of nucleotide changes per site. (**c**) Geographical distribution of *Ca*. Sivonenia alaskensis based on significant matches with metagenomic and 16S rRNA gene amplicon sequencing datasets in SRA (≥20 % containment and ≥0.1 % relative abundance, respectively).

We investigated in more detail the two *Gloeobacterales* MAGs ‘PMM_0042’ and ‘PMM_0068’ given the importance of this group as the most basal lineage of extant Cyanobacteria [97–100]. Phylogenetic analysis based on a concatenated alignment of 38 ribosomal proteins placed both MAGs alongside the MAG ‘IMG_3300025548_6’ [39] (Fig. 4a), with which they share 97.2 % ANI (Table S3). This is above the ≥95 % ANI threshold commonly used for delineating microbial species [63], which thus suggests that the three MAGs (‘PMM_0042’, ‘PMM_0068’ and ‘IMG_3300025548_6’) belong to the same species. Furthermore, their phylogenetic placement and low ANI (<80 %) with other *Gloeobacterales* indicate that they constitute a distinct genus in this order. Separation from the other *Gloeobacterale* s is also supported by analysis of the 16S rRNA gene of the MAG ‘IMG_3300025548_6’, which is 91.8–92.0 %, 90.0% and 89.3 % similar to the sequences of *Gloeobacter* spp., *Candidatus* Aurora vandensis and *Anthocerotibacter panamensis*, respectively (Fig. 4b). We consider that the MAGs ‘PMM_0042’, ‘PMM_0068’ and ‘IMG_3300025548_6’ represent a novel lineage in the order *Gloeobacterales* and propose the name *Candidatus* Sivonenia alaskensis (*Sivonenia*: in honour of our colleague and Cyanobacteria expert Dr Kaarina Sivonen, *professor emerita* of the University of Helsinki; *alaskensis*: relative to the geographical origin of the MAG ‘IMG_3300025548_6’, which is proposed here as the nomenclatural type for this species according to the SeqCode initiative [101]).

### *In silico* analysis indicates that *Ca*. Sivonenia alaskensis is a thylakoid-less cyanobacterium

Analysis of the protein-coding genes of the *Ca*. Sivonenia alaskensis MAGs revealed many similarities with other *Gloeobacterales *, thus supporting their phylogenetic placement within this order of early branching Cyanobacteria (Fig. 4ab). For instance, strains of *Gloeobacter* spp. and *Anthocerotibacter panamensis* differ significantly from other Cyanobacteria by the lack of thylakoid membranes and the presence of a reduced photosynthetic apparatus [102–105]. These traits are considered ancestral features of oxygenic photosynthesis given the basal position of *Gloeobacterales* in the evolution of Cyanobacteria and plastids [97–99]. Like the genomes of other *Gloeobacterales* [39, 105–108], the *Ca*. Sivonenia alaskensis MAGs seem to lack the genes for several subunits of the photosystems I (PsaI, PsaJ, PsaK and PsaX) and II (PsbY, PsbZ and Psb27), the circadian clock (KaiA, KaiB and KaiC) and the thylakoid membrane (SqdB and SqdX) (Fig. 3, Table S5). Moreover, similarly to *Anthocerotibacter panamensis* and *Ca*. Aurora vandensis but unlike *Gloeobacter* spp [105]., *Ca*. Sivonenia alaskensis seems to lack two subunits of the photosystem II (PsbM and PsbU) and the cytochrome *b6f* complex (PetM and PetN), and does not appear to contain any gene involved in the synthesis of phycoerythrin (Pec). Overall, *in silico* analysis of the proteome of *Ca*. Sivonenia alaskensis suggests that this lineage comprises organisms without thylakoid membranes and with a reduced photosynthetic machinery, both of which are the defining characteristics of the order *Gloeobacterales* [66]. Moreover, the predicted structure of the photosystem II, cytochrome *b6f* and phycobilisome machineries of *Ca*. Sivonenia alaskensis holds more similarities with *Anthocerotibacter panamensis* and *Ca*. Aurora vandensis than with *Gloeobacter* spp., supporting the evolutionary relationship inferred from the analysis of ribosomal proteins and the 16S rRNA gene (Fig. 4ab). Whether the unique characteristics of *Gloeobacter* spp. reflect the ancestral state of the phylum *Cyanobacteria* has been an open question in the study of the early evolution of this group for many decades [100]. The discovery of *Ca*. Sivonenia alaskensis, *Anthocerotibacter panamensis* [105] and *Ca*. Aurora vandensis [40] suggests that characteristics such as the lack of thylakoid membranes and a reduced photosynthetic apparatus are indeed a pervasive trait of the early branching *Gloeobacterales *.

### *Ca*. Sivonenia alaskensis is distributed across the cold biosphere

Read recruitment analysis revealed that the two *Ca*. Sivonenia alaskensis MAGs are found in four microbial mat samples from the sub-Antarctic (Macquarie Island and Marion Island) and Antarctic Peninsula (King George Island), where they constitute up to 1.0 % of the metagenomes (Table S4). To gain further insights into the ecology of *Ca*. Sivonenia alaskensis, we used *sourmash branchwater* [73, 74] to search metagenomic datasets in SRA for sequences matching the *Ca*. Sivonenia alaskensis MAGs. We also searched its 16S rRNA gene in amplicon sequencing datasets in SRA using *IMNGS* [75]. This extensive search, which included collectively *ca*. 1.3 million public datasets from around the globe, revealed sequences related to *Ca*. Sivonenia alaskensis in lakes, sediments and soils across many polar, sub-polar and alpine environments, and to a minor extent at lower latitude and non-alpine regions (Fig. 4c, Table S6). Sequences matching the *Ca*. Sivonenia alaskensis MAGs were particularly abundant (0.4–8.0 %) in amplicon sequencing datasets of active communities (that is, derived from RNA molecules) in the sediment of thermokarst lakes near Barrow (Alaska) [109], as well as in metagenomic datasets from the sediment of Lake Hill (St. Paul Island, Alaska) [110] (0.8–3.1 % of the reads). Interestingly, sequences matching the 16S rRNA gene of *Ca*. Sivonenia alaskensis were found in several datasets obtained from the gut microbiome of stickleback fishes (Actinopterygii: Gasterosteidae) and mayflies (Insecta: Ephemeroptera) [111–113].

Despite their importance for the study of the evolution of oxygenic photosynthesis, little is known about the ecology of the early branching *Gloeobacterales* compared to the other Cyanobacteria [88, 97, 105, 114]. *Gloeobacter *, which was for many decades the only described genus in this order, is typically found in low-light, wet rock habitats [102, 115, 116]. Amplicon sequencing studies have also reported 16S rRNA gene sequences loosely related to *Gloeobacter* spp. in Arctic [18, 22] and temperate [117] soil crusts, and in Arctic [83, 118] and Antarctic [119] microbial mats. The phylogenetic and ecological range of the order *Gloeobacterales* has expanded recently with the discovery of *Ca*. Aurora vandensis from Antarctic lakes [40], *A. panamensis* associated with a tropical bryophyte [105], and five MAGs from different ecosystems including the *Ca*. Sivonenia alaskensis MAG ‘IMG_3300025548_6’ recovered from Arctic peat soil [39]. Apart from *A. panamensis*, *Gloeobacterale* s appear to show a preference for low-light environments. This has been linked to their slow growth which, in turn, appears to be a consequence of their reduced photosynthetic apparatus [105]. Furthermore, our results suggest that *Ca*. Sivonenia alaskensis is distributed across cold regions, especially polar and alpine lakes and sediments (Fig. 4c, Table S6). Its high abundance in an RNA-derived amplicon sequencing dataset of lake sediments in Alaska [109] suggests that *Ca*. Sivonenia alaskensis forms active populations in this habitat. By contrast, the detection of sequences matching the 16S rRNA gene of *Ca*. Sivonenia alaskensis in the microbiome of stickleback fishes does not mean that they are active members of gut communities. These sequences probably represent cells that were ingested either incidentally or collaterally via zooplankton that is consumed by the fish (D. Bolnick, personal communication).

### Analysis of resistance mechanisms to environmental stress in *Ca*. Sivonenia alaskensis

To obtain insights regarding the distribution of *Ca*. Sivonenia alaskensis across the cold biosphere, we searched the MAGs for genes involved in resistance mechanisms to environmental stress. We found 75 genes related to mechanisms to cope with desiccation, cold and ultraviolet radiation (UVR) stresses in at least one of the *Ca*. Sivonenia alaskensis MAGs (Table S7). Among these are genes involved in the Wzy- and ABC transporter-dependent pathways for the assembly and export of extracellular polymeric substances (EPS). The production of an EPS matrix is a mechanism that is commonly employed by Cyanobacteria to cope with desiccation and freezing [120]. Genes involved in the synthase-dependent pathway of EPS production were not found. Scytonemin and mycosporine-like amino acids (MAAs) are often produced by Cyanobacteria as UVR-screening compounds [121]. Despite having several of the genes involved in the production of scytonemin and MAAs, the genes encoding the key proteins ScyC, ScyD, EboA, EboB, EboC and MysC were not found. As such, the production of these compounds by *Ca*. Sivonenia alaskensis is unlikely. We identified other mechanisms of resistance to cold in the *Ca*. Sivonenia alaskensis MAGs, including proteins involved in regulation of cell membrane fluidity, regulation of replication and translation, and RNA metabolism (Table S7). Although these proteins are part of the general cell functioning, the up-regulation of their genes has been reported as a cold-shock response in Cyanobacteria [122, 123]. Finally, mechanisms of DNA repair include the base excision repair pathway for several glycosylases, the homologous recombination pathway for single-stranded breaks and one of the subtypes of the nuclear excision repair pathway.

## Conclusion

We investigated 17 polar microbial mat metagenomes and recovered 37 MAGs of Cyanobacteria representing 17 species at different levels of phylogenetic novelty: around half of the MAGs are very distant (<80 % ANI) to genomes currently available in GenBank; the other half are related to polar and alpine strains with varying levels of genome similarity (80.1–99.8% ANI). Among the latter, we describe the phylogenetic, metabolic potential and ecological characteristics of a lineage in the early branching *Gloeobacterales *. *In silico* analyses indicate that this lineage – which we name *Ca*. Sivonenia alaskensis – is a thylakoid-less cyanobacterium that is found across cold environments and harbours common mechanisms of resistance to environmental stress. Our study shows that genome-resolved metagenomics is a reliable and straightforward way of recovering novel genomes of Cyanobacteria without the need for strain isolation. However, strain isolation is still useful for many purposes and may in fact benefit from genomic information obtained from MAGs to design protocols for targeted isolation. Based on the ≥95 % ANI threshold commonly used for delineating microbial species [63], most of the MAGs obtained represent different species or even genera from those currently represented by genomes in GenBank. Comparison with strains without genome data was not possible as only one of the 37 MAGs included the 16S rRNA gene, which is the most widely used molecular marker for the taxonomy of Cyanobacteria [28]. Assembling and binning 16S rRNA genes from short-read metagenomic data is difficult due to the highly conserved nature of this gene and its skewed coverage and sequence composition signals compared to the rest of the genome. The use of long-read technologies (e.g. Oxford Nanopore and PacBio SMRT sequencing) could help alleviate this issue [124, 125].

## Supplementary Data

Supplementary material 1
